# Freely Available Virtual Reality Experiences as Tools to Support Mental Health Therapy: a Systematic Scoping Review and Consensus Based Interdisciplinary Analysis

**DOI:** 10.1007/s41347-021-00214-6

**Published:** 2021-06-23

**Authors:** Paul Best, Matilde Meireles, Franziska Schroeder, Lorna Montgomery, Alan Maddock, Gavin Davidson, Karen Galway, David Trainor, Anne Campbell, Tom Van Daele

**Affiliations:** 1 School of Social Sciences, Education and Social Work, Queen’s University, Belfast, Northern Ireland; 2 Sonic Arts Research Centre (SARC), Queen’s University, Belfast, Northern Ireland; 3 School of Nursing and Midwifery, Queen’s University, Belfast, Northern Ireland; 4 Sentireal Ltd, Belfast, Northern Ireland; 5 Expertise Unit Psychology, Technology & Society, Thomas More University of Applied Sciences, Antwerp, Belgium; 6 The Immersive Technologies and Digital Mental Health Network, Queen’s University, Belfast, Northern Ireland

**Keywords:** Virtual reality, Mental health, Therapy, Scoping review, Freely available

## Abstract

The primary purpose of this article is to review the potential therapeutic value of freely available VR content as an addition to the practitioners ‘toolkit’. Research has shown that virtual reality (VR) may be useful to extend existing guided imagery-based practices found in traditional mental health therapy. However, the use of VR technology within routine mental health practice remains low, despite recent reductions in equipment costs. A systematic scoping review and interdisciplinary analysis of freely available VR experiences was performed across two popular online databases (SteamVR and Oculus.com). A total of 1785 experiences were retrieved and screened for relevance with 46 meeting the inclusion criteria. VR content was then reviewed for potential therapeutic value by an interdisciplinary panel with experience across a number of therapeutic interventions including cognitive behavioural therapy, Rogerian counselling, mindfulness-based therapies. and family therapy. Eleven (22%) of the 50 freely available VR experiences were reported to have therapeutic potential as tools to support routine mental health therapy. These included support with the following mental health issues—low mood, social anxiety, stress reduction and fear of heights. Guidance of a qualified mental health practitioner was recommended in all cases to maximise the benefit of the VR experiences retrieved. While the quality is variable, freely available VR experiences may contain valuable content that could support mental health therapy. This includes as a homework activity or as an initial setting for case formulation and behavioural experiments.

## Introduction

Over the past 25 years, there have been considerable developments in the use of virtual reality (VR) as a specialized tool for therapeutic purposes, in particular for the treatment of posttraumatic stress disorder (PTSD) (Best et al., 2020; Deng et al., 2019) and specific phobias (Botella et al., 2017). In fact, a recent systematic ‘review of reviews’ found that there was evidence supporting the positive impact of VR and notably that no paper “has concluded that VR does not work” for psychiatric disorders (Cieślik et al., 2020: 13). There do seem to be many inherent features that make VR ideally suited for mental health therapy. These include the use of imagery to stimulate cognitive, emotional and physical reactions—much in the same way, therapists traditionally use imagery (such as still images or visualisation) or various image re-scripting techniques in order to elicit and address problems in their clients’ life (Holmes et al., 2007; Hales et al., 2015). Previous work by Rizzo et al., (2004; 2019) and Riva et al. (2004) went further, outlining a number of intrinsic features of VR technology and their suitability within mental health therapy, such as opportunities for live performance monitoring, tailored exposure and the ability to recreate real-world situations that may otherwise be risky. Research on the potential of VR for mental health practice has continued to grow over the past decades (Valmaggia et al., 2016) and expanded into other areas, such as tailoring VR environments to the treatment of pain (Jerdan et al., 2018; Mallari et al., 2019), eating disorders (Clus et al., 2018) and psychosis (Rus-Calafell et al., 2018).

In recent years, the commercialization of VR technology has significantly increased availability and reduced costs, both in terms of hardware (headsets) and software applications (experiences), to make use of VR in mental health practice (Bun et al., 2017). Simultaneously, a limited but growing audience has had the chance for a first-hand experience of VR (Vandendriessche & De Marez, 2020). The majority of VR apps however do not have a clinical focus and are instead being developed for entertainment purposes (Newby and Jiang, 2018). Those who do have a clinical focus have often not been evaluated, although some have been developed according to evidence-based principles (Freeman et al., 2017). Even more uncommon is a stand-alone experience with demonstrated effectiveness that is commercially available. A rare example is ZeroPhobia, a VR experience that is accessible on a smart phone and is based on conquering a fear of heights, has demonstrated effectiveness in a waitlist RCT for acrophobic clients (Donker et al., 2019).

While this illustrates that a sufficient evidence-base for VR experiences can be achieved, the programme is only available when paying a fee. More extensive VR packages are available as well. These allow practitioners to rely on a large variety of environments and to integrate with a wider range of services (e.g., Psious, OxfordVR, Mimerse). Again, however, most are fee paying, and only a relatively small number are available through VR app stores, such as SteamVR and Oculus.com, making accessibility an issue. Previous studies have shown that practitioners do have a generally positive attitude towards the idea of using technology within practice (Cliffe et al., 2020; Stallard et al., 2010). Even for VR, which was often perceived as technologically inaccessible and expensive, attitudes have shifted and currently seem more favourable (Lindner et al., 2019). Unfortunately, those who are willing to take the leap and integrate technology in their practice are often faced with challenges and barriers concerning the cost of paid applications, as healthcare systems struggle to determine adequate means of reimbursement (Powell et al., 2019). These struggles are detrimental for the dissemination and uptake of paid, high-quality applications and platforms. Research on smartphone app usage in the general population has already shown that the average consumer is not too eager to pay for applications (Dinsmore et al., 2017). This is clearly reflected by the fact that three quarters of general purpose apps are no longer being monetized by charging a fee in exchange for download. Increasingly, applications are opting for other monetization models like in-app advertising or in-app purchases of additional content and features (Dinsmore et al., 2017). Such practices might, however, not be easily transferred to a healthcare context.

Overall, the combination of a relative lack of evidence-based applications and the reluctance to pay for services creates a sub-optimal context to stimulate short-term uptake of VR. The few documented cases in routine practice nevertheless do show promise (Lindner et al. 2019). In anticipation of continued policy-level changes towards the use of technology that might stimulate the use of VR for therapeutic purposes, the current paper sets out findings from a systematic scoping review and interdisciplinary analysis to identify and appraise the therapeutic potential of freely available VR experiences across different platforms and devices. The aim is to illustrate which VR-based tools are available and for which purposes these might be relevant.

### Objectives

- To determine the number of freely available VR experiences which may have relevance to mental health
- To conduct an initial quality appraisal of freely available VR content relevant to mental health
- To conduct a consensus-based, interdisciplinary analysis of freely available (categories of) VR experiences to discuss potential therapeutic potential

## Methodology

The study design is novel and specific to reviewing VR content. It is informed by approaches to systematic scoping reviews and consensus building methods in healthcare (Arksey & O’Malley, 2005; Best et al., 2014; Fink et al., 1984; Halcomb et al., 2008; Murphy et al., 1998; Taylor et al., 2003). By combining key aspects of both approaches, the following core activities were undertaken:

1. The selection of an interdisciplinary panel with defined specialities or relevant professional backgrounds
2. The identification of relevant VR experiences using systematic searching techniques
3. The preparation of graded material provided to panel members in advance of meetings
4. Opportunity for discussion and consensus building through group and individual meetings

### (1) The Selection of an Interdisciplinary Panel with Defined Specialities or Relevant Professional Backgrounds

An interdisciplinary panel of reviewers was recruited that could assess the features of VR experiences from both a technical and clinical standpoint. In total, 10 reviewers (5 males and 5 females) took part. This included those with professional qualifications and experience in clinical psychology, mental health social work, cognitive behavioural therapy, humanistic counselling, systematic family therapy, mindfulness-based therapies and spatial and immersive audio. A unique aspect of the study was the inclusion of those with technical expertise on the review panel. This included those with experience of developing both the visual and audio-based components of virtual reality environments. This is a key factor given that data regarding the immersive quality of VR experiences is often best described in more objective terms, such as how well external sensory information is blocked.

### (2) The Identification of Relevant VR Experiences Using Systematic Searching Techniques

Given the volume of freely available VR content, the amount of material provided for interdisciplinary discussion was screened and reduced according to project exclusion and inclusion criteria (see below). In order to achieve this, a systematic search was conducted in two popular VR databases (SteamVR and Oculus.com). Primary searching took place over a 3-week period in September 2019 and was updated in January 2021 and involved 24 keywords related to mental health (Table 1).

**Table 1:** List of key words

Anxiety	Mindfulness
Fear	Cognitive behavioural therapy
Trauma	Counselling
Social anxiety	Exposure
Social phobia	Phobia
OCD	Therapy
Panic	Well-being
Relax	Disorder
Meditation	Mood
Scared	
Scary	
Intense	
Mental health	
Calm	
General anxiety	

**Fig. 1:**
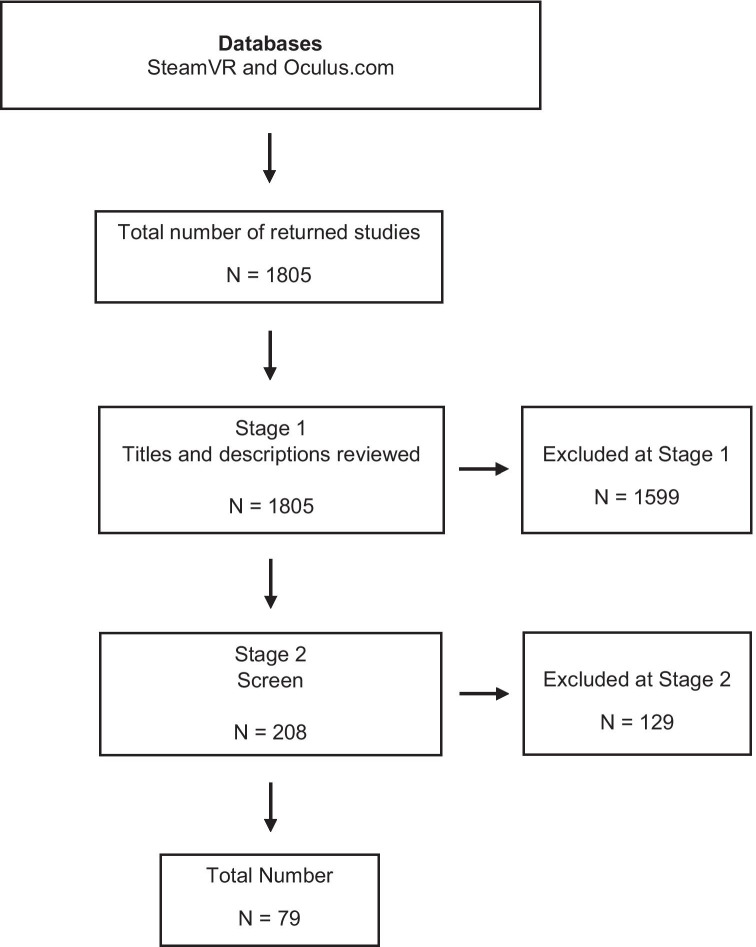
Overview of search strategy

Overall, 1805 VR experiences were retrieved and initially screened via title and description by MM. At this initial stage, the main inclusion criteria were (1) is the VR content freely available (in-part or in full) and (2) is there a suggested or implied mental health focus, benefit or response. Following the removal of duplicates, fee-paying experiences and those that did not meet initial criteria, a total of 208 VR experiences went forward to the next stage of screening. During this second stage, the reviewers went into more depth by reviewing video trailers and pictures (if available) associated with all 208 VR experiences as well as message boards and user reviews available on both SteamVR and Oculus.com platforms. It was also verified that applications which presented themselves as freely available were indeed accessible at no expense. As a result, the final number of experiences selected for full review and appraisal was 79 (Fig. 1).

### (3) The Preparation of Graded Material Provided to Panel Members in Advance of Meetings

In order to grade and synthesise data from all 79 VR experiences, a bespoke appraisal tool was developed and administered using Google Forms (see Appendix). The appraisal tool included the following Sects. (1) description of VR experience, (2) screening items, (3) four core domains, and (4) a qualitative comment(s) section. In order to increase the robustness of the appraisal process, each VR experience received two independent reviews and at least one of the reviewers had to have a mental health background. Reviewers were assigned an equal number of experiences to view and given access to the appropriate equipment (headset) to complete the appraisal. Scores were then collated to give a mean overall. The internal consistency of this measurement was tested using a Cronbach’s alpha test of reliability, which deems that any scale measurement which is 0.7 or over is internally consistent (Cortina, 1993).

#### Screening Items

 Four screening items were included within the appraisal tool. If ‘No’ was recorded against the first two items, then the review would not proceed. These questions were as follows *(a) based on the manufacturer description, is the experience Mental Health focussed? and (b) If no, could the content potential be indirectly used for mental health exposure or treatment?* The final two items were designed to streamline the review process and to broadly categorise each experience in relation to a potential condition and treatment approach/technique. For example, if a VR experience contained content that may have related to a specific phobia, then it would be broadly categorised as ‘exposure’. If the content was focused on stress reduction or in included elements of guided relaxation, then it was broadly categorised as ‘relaxation/meditation’.

#### Domain 1: Emotional/Physical Response

 This section ascertained the level of sensory experience (emotional or physical) that the programme was able to generate within the user. This can be positive (relaxing, calming) or negative (fear, apprehension). Reviewers were asked to rate both the emotional and physical shifts generated through the VR experience using a 5-point Likert scale ranging from 1 ‘none’ to 5 ‘substantial’. A total score between 2 and 10 was therefore generated. In case of an insufficient shift in reported sensations (a score below 6), no further appraisal was conducted. The Cronbach’s alpha for this 2-item measure was .79.

#### Domain 2: Immersive Experience

 This section focused on the immersive aspects of the VR experience and was developed from items within the immersive experiences questionnaire (Jennett et al., 2008). Reviewers were asked to appraise how well the VR experience blocked out real-world sensory information through graphic quality and immersive audio. Immersion is achieved by removing as many of the real-world sensory information as possible, and thus, the focus here was on how well the technology is able to do this. Again, 5-point Likert scales were used across three items, ranging from 1 ‘poor’ to 5 ‘excellent’, giving scores ranging from 3 to 15. The Cronbach’s alpha for this 3-item measure was .79.

#### Domain 3: Assets

 This section was based on the work of Rizzo et al., (2004; 2019) and Riva et al. (2004) who describe the various features of VR that may be beneficial within therapeutic contexts (user control, opportunity for feedback, system interaction etc.). As such, a checklist was developed in which the reviewer was invited to state which assets/affordances were present within each VR experience. These items were designed to draw the reviewer’s attention to the potential value of the experiences within their practice (in their current form). As this is a checklist rather than a scale, no reliability analysis was undertaken.

#### Domain 4: Presence:

 Presence is defined as a user’s subjective sensation of “being there” (Barfield et al., 1995). This section used the 14-item IGroup Presence Questionnaire (IPQ; Schubert, 2003), which has been validated across multiple samples. Presence is often associated with the overall quality of a VR experience, and as such, this validated measure was an important indicator that the research team sought to incorporate. The Cronbach’s alpha for the present study was .87.

In addition to this, the appraisal tool had an initial set of descriptor questions regarding the date accessed, the database where the experience was located and comfort level of the experience (comfortable, moderately intense, intense, unrated). A qualitative comments section was also included as a final section to capture any information that was not specifically addressed within the tool. It is important to note that the data generated during this stage was intended to inform interdisciplinary panel discussion and not to pre-determine any findings. As such, panel members were still encouraged to further scrutinise all of the data presented to them.

### (4) Opportunity for Discussion and Consensus Building Through Group and Individual Meetings

The final activity involved presenting the graded and synthesised results to an interdisciplinary panel for in-depth discussion and review that had took part in initial appraisals. Due to COVID-19 restrictions, panel discussions were conducted via Microsoft Teams with each member having access to a VR Headset to revisit experiences if required. Panel members were asked to discuss the therapeutic value of each VR experience and to indicate for which condition they might be relevant. The ultimate goal of this activity was to produce a revised table and ranking system following in-depth interdisciplinary discussion.

### Data Analysis

Data analysis had two distinct stages. Stage 1 involved analysing the 148 independent reviews (two reviews per experience) using SPSS v24 (Pallant, 2004) by combining them across each of the four domains to produce an overall mean score. In order for comparison across scales—mean scores, standard deviations and scatterplots were used to produce four board classifications within each domain—(1) very low; (2) low (3) moderate and (4) high. As the primary goal was to inform interdisciplinary discussion, no further statistical tests were performed. At stage 2, analysis was conducted through interdisciplinary discussion and debate in order to reach a consensus. Key questions that informed the analysis were—(1) what mental health condition or issue might be relevant for this VR experience, (2) what key features are associated with this experience and (3) how feasible is it to implement within a routine mental healthcare setting or treatment protocol?

## Results

### Stage 1: Appraisal Tool Scores—Initial Grading and Synthesis

Of the 79 VR experiences reviewed, 50 passed initial screening and were selected to proceed to interdisciplinary review. Table 2 shows the ranking of all 50 VR experiences based on collated scores following stage 1 analysis. The majority of the experiences were available on the Oculus Go (*n* = 27, 54%), followed by HTC Vive (*n* = 14, 28%), Oculus Rift (*n* = 8, 16%), Oculus Quest (*n* = 2, 4%) and Gear VR (*n* = 1, 2%). The potential therapeutic value of the VR experiences was most often categorised as exposure based (*n* = 22), followed by relaxation/meditation (*n* = 22), immersive story telling (*n* = 3), psychoeducational (*n* = 2) and peer support (*n* = 1). In terms of the ability to generate emotional or physical responses, 4 (8%) experiences were rated as ‘high’, with 15 (30%) as ‘moderate’, 12 (24%) as ‘low’ and 20 (40%) as ‘very low’. Potential features which may be useful in therapeutic contexts were represented by total ‘assets’ score. This was rated high in 8 (16%) VR experiences whereas 16 (32%) achieved an assets rating of moderate followed by 13 (26%) rated as low and 9 (18%) rated as very low.

**Table 2:** Full list of VR appraisal scores at stage 1

	Title^a^	Type	Emotional/physical response^b^	Immersion	Presence	Assets	Headset	Appraisal score
1.	Face your fears—Killer view	Exposure	High	High	Moderate	Moderate	Oculus Go/Rift	14
2.	Happy Place	Relaxation/meditation	Moderate	Moderate	High	High	Oculus Go	14
2.	Liminal	Relaxation/meditation	High	Moderate	Moderate	High	Oculus Go	14
3.	TRIPP	Relaxation/meditation	Moderate	High	High	Moderate	Oculus Quest	14
4.	Alcove	Relaxation/meditation	Low	High	High	High	Oculus Quest	14
5.	Binaural Odyssey	Relaxation/meditation	Low	High	High	Moderate	HTC Vive	13
6.	Dreadhills Demo	Exposure	Moderate	Moderate	High	Moderate	Oculus Go	13
7.	Self-knowledge VR	Psycho-educational/self-awareness	Low	High	Moderate	High	HTC vive	13
8.	Breath Peace World	Relaxation/meditation	High	High	Very low	Moderate	Oculus Go	12
9.	Bridge Trek	Relaxation/meditation	Moderate	High	Low	Moderate	Oculus Rift	12
10.	Calm Place	Relaxation/meditation	Moderate	Moderate	Moderate	Moderate	Oculus Go	12
11.	Fear of Public Speaking—Business Life	Exposure	Moderate	Low	Moderate	High	Oculus Go	12
12.	Fear of Public Speaking—School Life	Exposure	Low	Low	High	High	Oculus Go	12
13.	Gogglebox	Exposure	Moderate	Moderate	Moderate	Moderate	Oculus Go	12
14.	Hehu and the Taniwha	Exposure	Moderate	Moderate	Moderate	Moderate	Oculus Rift	12
15.	Rec room	Exposure	Very low	High	High	Moderate	Oculus Rift	12
16.	VR Jogger	Exposure	Moderate	Low	High	Moderate	Oculus Rift	12
17.	Calm	Relaxation/Meditation	Moderate	Moderate	Moderate	Low	Oculus Go	11
18.	Dances with Butterflies VR	Relaxation/meditation	Very low	Moderate	Moderate	High	HTC Vive	11
19.	Guided Meditation VR	Relaxation/meditation	Moderate	Low	Moderate	Moderate	Oculus Go	11
20.	Insanity VR	Exposure	Moderate	High	Low	Low	HTC Vive	11
21.	RCSI Medical Training Sim	Psycho-educational/exposure	Moderate	Low	Moderate	Moderate	Oculus Go	11
22.	RideOp	Exposure	Moderate	Low	High	Low	HTC Vive	11
23.	Grove VR	Peer support	Low	Low	Moderate	Moderate	Oculus Go	10
24.	Being a Bystander	Exposure	Moderate	Low	Moderate	Low	Oculus Go	10
25.	House of Terror	Exposure	High	Low	Very low	Moderate	Oculus Go	10
26.	Story UP	Immersive story telling	Low	Moderate	Moderate	Low	Oculus Go	10
27.	Purgation	Exposure	Moderate	Low	High	Very low	HTC Vive	10
28.	Clean VR	Exposure	Very low	Low	Moderate	Moderate	HTC Vive	9
29.	Fear of heights—Cityscapes	Exposure	Low	Low	Very low	High	Gear VR	9
30.	Fear of Public Speaking—Personal Life	Exposure	Very low	Low	Low	High	Oculus Go	9
31.	Kinese	Relaxation/meditation	Very low	Low	High	Low	HTC Vive	9
32.	LetB	Relaxation/meditation	Low	Moderate	Low	Low	Oculus Go	9
33.	The Bellows	Exposure	Very low	High	Low	Low	HTC Vive	9
34.	BeFearless Fear of Heights Landscapes	Exposure	Low	Very low	Low	Moderate	Oculus Go	8
35.	Letzte Worte VR	Immersive story telling	Low	Low	Moderate	Very low	Oculus Rift	8
36.	Mindverse	Relaxation/meditation	Low	Very low	High	Very low	Oculus Rift	8
37.	Stargate Media	Exposure	Low	Low	Low	Low	Oculus Go	8
38.	VRChat	Exposure	Very low	Low	Moderate	Low	Oculus Rift	8
39.	VR Retreat	VR Retreat	Very low	Low	Low	Moderate	HTC Vive	8
40.	Guided Relaxation VR	Relaxation/meditation	Low	Low	Very low	Low	Oculus Go	7
41.	Inevitable VR	Exposure	Very low	Moderate	Low	Very low	HTC Vive	7
42.	Shinrin-yoku: Forest Meditation	Relaxation/meditation	Very low	Moderate	Very low	Low	HTC Vive	7
43.	Speech Trainer	Exposure	Very low	Very low	High	Very low	HTC Vive	7
44.	SZEN	Relaxation/meditation	Very low	Low	Moderate	Very low	HTC Vive	7
45.	Ozential	Relaxation/meditation	Low	Low	Very Low	Very Low	Oculus Go	6
46.	Helium Stories for Muse	Relaxation/meditation	Very low	Low	Very low	Low	Oculus Go	6
47	Tiny Island relax	Relaxation/meditation	Very low	Very low	Very low	Moderate	Oculus Go	6
48.	Dream	Relaxation/meditation	Very low	Low	Very low	Very low	Oculus Go	5
49.	The Ancient Island	Relaxation/meditation	Very low	Low	Very low	Very low	Oculus Go	5
50.	VR Church: The Bible	Immersive story telling	Very low	Very low	Very low	Very low	Oculus Go	4

^a^When appraisals scores were identical, experiences were listed in alphabetical order

^b^High = 4, moderate = 3, low = 2 and very low = 1

### Stage 2: Interdisciplinary Discussion and Consensus Building

Using the information provided during stage 1 analysis, the panel quickly determined that a number of VR experiences (*n* = 7) were survival/horror based and should be discounted as they offered little therapeutic value. It was also considered that VR experiences designed to simply ‘shock’ or surprise may have inflated scores in the emotional and physical response domain of the appraisal tool and should be interpreted with caution. For example, *‘Fear your Fears—Killer View’* is an experience designed to evoke a fear of heights among its users. While panel members agreed that the programme was successful in triggering a range of somatic responses associated with a fear of heights, it was decided that the experience was too intense and insufficiently controllable to be used within mental healthcare settings for acrophobia clients. Moreover, the experience was relatively short, and there was little opportunity for the therapist (or user) to control the level of exposure (e.g. by pausing the scenario). As such, habituation would have been difficult to achieve. Other experiences, such as Speech Trainor, appeared to have clear relevance for public speaking anxiety and included interesting features, such as the ability to upload content (PowerPoint presentations) and hold a microphone. However, issues regarding intermittent lag in audio and limited audience reaction meant that immersive quality was impacted. The panel also queried the categorisation of some experiences, such as *Stargate Media* as having exposure-based components and whether others, such as *Bridge Trek*, could be a useful setting for both the application of exposure-based techniques as well as a place for relaxation. Consequently, the panel determined that of the 50 freely available VR experiences presented to them, only 11 (22%) had the potential to support mental healthcare practice (Table 3).

**Table 3:** VR Experiences with potential therapeutic value

	Name	Mental health issue	Link
1	Liminal	Low mood and stress reduction	https://liminalvr.com/
2	Bridge Trek	Fear of heights and (general) anxiety	https://store.steampowered.com/app/749180/Bridge_Trek/
3	Rec room	Social anxiety	https://store.steampowered.com/app/471710/Rec_Room/
4	Calm Place	Anxiety and stress reduction	https://mimerse.com/products/calm-place/
5	Breath Peace World	Anxiety and stress reduction	https://www.oculus.com/experiences/rift/1,526,202,524,057,260/?locale=en_GB
6	Happy Place	Anxiety and stress reduction	https://www.oculus.com/experiences/gearvr/1064866736899927/?locale=en_GB
7	Fear of Public Speaking—Business Life	Fear of public speaking and social anxiety	https://www.oculus.com/experiences/gearvr/942681562482500/?locale=en_GB
8	GroveVR	Peer support	https://www.grovevr.com/
9	TRIPP	Low mood and stress reduction	https://www.oculus.com/experiences/quest/2173576192720129/?locale=en_GB
10	Alcove	Low mood and stress reduction	https://www.oculus.com/experiences/quest/3895528293794893/?ranking_trace=0_3895528293794893_SKYLINEWEBQUESTSEARCH_1buhKPYK6Unzg0NE2
11	Binaural Odyssey	Anxiety and stress reduction	https://store.steampowered.com/app/1421100/Binaural_Odyssey/

All 11 VR experiences were viewed as an adjunct, rather than replacement, to face-to-face mental health therapy (e.g. homework activity or as part of graded exposure experiments). The panel also concluded that the therapeutic value of some VR experiences may only be achieved under strict supervision of an appropriately trained mental health practitioner. For example, discussions regarding *REC Room* as an experience to support those with Social Anxiety Disorder were based on the premise that a qualified practitioner could use this experience as a tool within the early stages of therapy to build confidence or elicit key cognitions or emotions during initial assessment. The following section summarises experiences contained across each mental health issue as determined by the panel, including suggestions for potential use within practice.

### Low Mood and Stress Reduction

#### Liminal

According to its creators, Liminal is designed to ‘induce and augment emotional and cognitive states’ with experiences designed around four main categories: calm, energy, awe and pain relief. Before entering each experience, Liminal provides a brief description of the content as well as previous user scores and information on the developer(s). Users are prompted to indicate their current emotional state (e.g. neutral, anxious or sad etc.) before and after each activity, and design of the interface was intuitive. The graphic and audio quality of content within Liminal was considered basic, but panel members and reviewer scores suggested that it did not detract from the overall experience. A prompt at the beginning encourages the use of headphones to get the best experience.

In terms of therapeutic value, panel members highlighted the potential of Liminal to support those experiencing symptoms of anxiety, stress and low mood. For example, Liminal features a number of attention regulation tasks, such as focused breathing through 3D rings, throwing and controlling a lantern or catching butterflies in a jar. This is supported by either relaxing or energising music contained within a colourful virtual backdrop. As attentional and emotional regulation techniques are used in the treatment of a number of anxiety disorders, including generalised anxiety disorder and social anxiety disorder (Goldin et al., 2009; Renna et al., 2018). Liminal may offer some benefits for this client group.

In addition, Liminal introduces components of mindfulness-based practice (e.g. breath awareness, mindful movement etc.) which have been shown to improve depressive symptoms (MacKenzie et al., 2018). The panel also considered how tasks contained within the ‘energy’ category (e.g. time pressured challenges) could be useful as distraction exercises or as part of a wider behavioural activation (BA) approach for low mood. As Powell (2008) states, ‘individuals with depression often withdraw from positive activities and experiences’ (p. 78). BA is used to help individuals change how they feel by helping them change what they do. By doing so, they increase one’s access to positive reinforcement (Martell et al., 2010). In the case of Liminal, this may be through achieving goals or completing a challenge within a certain time. The panel agreed that Liminal could be used as a low-level, standalone VR experience for anxiety, stress and low mood. However, in order to gain maximum value, this experience would be best suited as an adjunct to face-to-face therapy—perhaps as a homework activity.

Other VR experiences in this category were TRIPP and Alcove. TRIPP is advertised for reducing stress and building resilience. It includes an 8-min demo experience whereby relaxation is encouraged through controlled breathing exercises (breathing in and out beams of light) as well as distraction via a short mini game in which you control an object with your gaze to navigate past oncoming boulders and collect coins. The panel rated the graphic and audio quality within TRIPP highly but noted the majority of content was hidden behind a paywall so the experience is somewhat limited when compared to Liminal. Alcove VR is described as a ‘virtual home’ where one can connect with friends and family to play games, relax and explore locations from across the globe. The panel felt the relaxation area within Alcove may be useful given the content related to meditation, relaxation as well as exercise. Alcove VR was discussed mainly in the context of a virtual ‘getaway’ that clients could visit when stressed rather than a tool to be used within a therapy session.

### Fear of Heights and (General) Anxiety

#### Bridge Trek

Bridge Trek is described as a safe environment in which one can conquer issues, such as fear of bridges or fear of heights in VR. The virtual environment includes a rooftop garden area with bridges of different heights as well as ladders, fish ponds and various fauna. There are no directed tasks, activities or audio narration within Bridge Trek, and users are free to explore the environment at their own pace and level of comfort. The user interface is intuitive, and there is no detailed menu system in order to access the experience. The potential therapeutic value offered by the Bridge Trek was discussed in relation to both the application of exposure-based techniques (anxiety/phobia) as well as an environment in which one could practise meditation or relaxation (for low mood and anxiety).

In regard to exposure, panel members felt that Bridge Trek included a range of graded activities for acrophobia that could prove useful as part of a therapeutic intervention. Bridges within Bridge Trek are placed at a number of different heights and include a range of designs (e.g. wood and glass). A client could be encouraged to walk over each height until their anxiety response has reduced by at least 50% using subjective units of distress (SUDs)—thus encouraging habituation. An important feature of Bridge Trek is that the user is in control of the experience and can choose the order in which to attempt each bridge which fits well within a graded exposure approach. While opportunities for synchronous communication (with a practitioner) were limited during the experience, the panel felt that discussions could take place between beforehand regarding the order in which certain bridges could be undertaken.

In regard to relaxation and meditation, panel members described Bridge Trek as providing a pleasant visual and auditory experience that would assist one to perform a number of relaxation techniques, such as focused breathing and body scanning. Moreover, the experience may be of benefit for those who struggle with visualisation during meditative exercises. As such, Bridge Trek may be useful as a daily meditative homework activity. The panel did note that while freedom afforded with Bridge Trek was beneficial, there were no audio-guided meditation or relaxation features contained within the programme. Moreover, there was no opportunity to track or record one’s progress over time which would have been particularly useful for graded exposure exercises.

### Social Anxiety

#### Rec Room

Rec Room is a VR-based social environment which, according to its developers, one can ‘hang out with friends from all around the world’. Environments within Rec Room can also be custom built by users to which they can invite others. This VR experience has no upper or lower age limits but seems primarily aimed at a younger person audience. The user interface in Rec Room is initiative, and visual graphics are basic but colourful. Given that REC Room appears an extension of the traditional internet chat room experience, the panel discussed its relevance for treating conditions, such as social anxiety disorder (SAD).

SAD refers to the persistent fear and/or avoidance of social situations related to the possibility of scrutiny by others and fears of acting in such a way that is embarrassing or humiliating (APA, 2013). In their cognitive model for SAD, Clark and Wells (1995) suggest that individuals with SAD make a series of negative ‘assumptions about themselves and the social world’ (Clark, 2001, p. 406) which can be divided into three main categories—(1) excessively high standards for social performance; (2) conditional beliefs concerning the consequences of performing in a certain ways; (3) unconditional negative beliefs about the self. As such, treatment includes a number of behavioural and cognitive strategies to undermine or challenge these negative assumptions.

Rec Room may assist in the treatment of SAD by providing initial opportunities to explore and test the strength of these negative assumptions or beliefs experienced by those with SAD. This may involve initiating conversations with strangers or groups, experimenting and reacting to negative feedback or observing and modelling the actions of others. For example, the therapist may deliberately make some conversational or social missteps and have the client observe the reaction of others. A major benefit of Rec Room is the high assets score whereby the features inherent within the experience offer the user a sense of control (e.g. social interactions can be graded through number of people and length of interaction). It was also considered that as the therapist could be present within virtual space at the same time as the client, this would provide opportunity for detailed feedback and support. Panel members also suggested acceptability among users may be higher (initially) when compared to real-life interactions, and this might enable treatment to progress at a faster pace. Parallels were made with the high levels of acceptability for VR-based interventions among other disorders, such as PTSD (Loucks et al., 2019).

Some concerns however were raised as regards difficulties with online communication, notably that other users may react in ways that they would not in real life. This may have consequences for challenging negative assumptions and generalising them into a real-world social setting. In addition, the audio quality appeared to vary and was dependent on the equipment being used by each online user.

### Anxiety and Stress Reduction

#### Calm Place

Calm Place is described as ‘a virtual space for tranquility, relaxation and guided meditation’ that, according to the promotional material provided, is targeted at reducing stress and anxiety. There are three landscapes to choose from within Calm Place (forest/lake; sandy beach and; mountainous area), and users may also select from three intervention types including (1) guided relaxation, (2) mindfulness or (3) ‘enjoy nature’ which encourages one to focus on naturally occurring sounds within the environment. Each area and intervention is selected through an intuitive menu system which facilitates ease of navigation. Additional customisable options include setting the duration of the experience (e.g. 9–20 min), sound options (music and natural sounds), weather (rain or sunshine) and changing from daytime to night-time. Calm Place uses emoticons to help users record how they are feeling, and there are also options to for seated, standing or floor viewing positions. A rewards section also helps the user track their progress with badges earned for completing courses and time spent in sessions. The graphical and audio quality within Calm Place was described as basic with panel members viewing the design of each virtual environment as minimalist in terms of content and animation. This was in contrast with ‘Happy Place’, a similar free-to-play experience developed by the same company (mimerse.com) which featured more interactive content despite less customisable features.

In regard to the potential of Calm Place to reduce stress and anxiety, panel members drew some parallels with *Bridge Trek* in terms of providing a peaceful environment to practise relaxation and meditative experiences through supported visualisation. These techniques have all been shown to reduce stress and anxiety in previous research (Coppola & Spector, 2009; Grossman et al., 2004). However, Calm Place differentiates from Bridge Trek through its customisable features and options for guided interventions. For example, there are separate courses on mindfulness and relaxation as well as interactive graphics to assist with activities, such as controlled breathing. Therefore, Calm Place offers a more holistic experience where the user can choose to self-direct or be guided through the programme. The panel discussed Calm Place as a potential adjunct to therapy whereby users could practise relaxation, meditation or mindfulness-based activities within a home setting. One note of caution was raised in relation to the privacy settings within Calm Place whereby mood data is collected by the developers. The user can only disable this feature through the ‘privacy settings’ located in the settings menu.

Other VR experiences relating to anxiety and stress reduction were *Happy Place*, *Breath Peace World and Binaural Odyssey*. Happy Place was created by the same developers that produced Calm Place (described below), and this experience centred upon a quiet lake at the edge of a forest and mountainous range. Imagery is colourful and vibrant, with some opportunity to interact with the environment by directing your glaze at certain objects or spaces to trigger a brief animation. Happy Place includes options for guided audio narration and scenery alternates between day and night. Breath Peace World is similar in its use of colourful and vibrant imagery; however, the landscape is a snow-covered forest. The focal point within Breath Peace World is a small bear that encourages slow and controlled breathing through the movement of surrounding trees and a small sparkling light on its body. Given the animations involved, Breath Peace World was discussed as being particularly well suited for younger children. Overall, the experience was considered fairly intuitive albeit brief, and there was a distinct lack of additional features and functionality found in other similar experiences. Binaural Odyssey was available to download from Steam and is described as a ‘virtual world [that] reacts to the eyes of the viewer and creates visual shapes from whichever direction the user looks at’. There are no controls or detailed menu system in Binaural Odyssey and yet the experience is intuitive and easy to understand. Each experience within Binaural Odyssey is unique as new patterns emerge with changing audio as users’ gaze moves around the environment. The panel believed this experience would be a useful tool for anxiety and stress reduction, serving as both a calming and relaxing space while offering peaceful distraction.

### Fear of Public Speaking and Social Anxiety

#### Samsung #BeFearless Fear of Public Speaking

The Samsung BeFearless range includes three different VR experiences which all follow a similar format (school, business and personal life). For the most part, these experiences use virtual environments with some additional 360 video footage included as bonus material in some experiences (e.g. School Life). At the time of panel review, only the ‘business life’ experience was still available for download and thus is the primarily focus. This experience is designed to help ‘overcome your fear of public speaking’. It includes five virtual scenarios (job interview, business lunch management presentation, team meeting and job fair) where one is tasked with speaking and answering questions as directed by virtual characters. According to Samsung, the experience is designed to respond to the user performance by collecting data on voice volume, speaking pace eye contact and heart rate (the latter needs to link to a Gear S device). As a user ‘passes’ each scenario, more scenarios are unlocked.

As this experience provides the user with the opportunity to practise conversation skills within a ‘safe’ social setting and to receive feedback on performance, the panel discussed its value as a tool to support the treatment of social anxiety disorder (SAD). The usefulness of BeFearless Fear of Public Speaking as a tool to build confidence before undertaking some real-world experiments was discussed in a similar fashion to Rec Room; however, there were some notable differences. BeFearless utilises AI-supported computed generated characters in order to mimic real-life responses and feedback on user performance. While this provides a safer and more controlled environment, the responses are limited to non-verbal gesturing (nodding and shaking of head, looking at watch, coughing etc.). As such, the interaction is largely one way. What BeFearless does offer is a much more sophisticated (if at times unreliable) user analytics system in which objective data can be collected and fed back to the user. This is through a combination of self-report data on performance as well as changing virtual avatar behaviour based on objective user data, such as eye contact, heart monitor, speech recognition etc. This may provide valuable early information regarding key cognitions, emotions or behaviours that take place during social interaction that may help inform early case conceptualisation.

While the panel noted that Samsung #BeFearless Fear of Public Speaking appeared to have a more advanced level of AI when compared to other freely available VR experiences, it did not always work as expected. A number of panel members suggested that virtual characters did not always react appropriately in line with a change in performance and that the system could be easily manipulated into giving higher scores. This may have accounted for the lower score within the immersion category during stage 1 appraisals. Regardless of this, the panel felt that it may be of some therapeutic value in the initial stages of therapy or as part of an initial assessment of difficulties.

### Peer Support

#### Grove VR

Grove VR takes a peer support–based model and hosts it in VR. Once registered, users are given a short introduction by the developers and gain access to a virtual lobby where they can sign up to attend pre-existing support groups or create their own. Graphic and audio quality was considered basic but acceptable within the wider context of the experience.

Group size on Grove VR ranged from 3 to 6 members and current groups included a variety of mental health topics, such as depression and anxiety, panic attacks and addictions. Some groups were also based around specific themes, such as COVID-19, advice and support for fathers and infidelity support. By walking to the notice board, the user could view group start times as well as the number of spaces available. Each group has a nominated ‘host’, and those seeking to join must send a request in order to be invited. As such, this allows some control and moderation of who can attend. Panel members also commented positively on some of the security and privacy features of Grove VR. For example, Grove VR required a pin code each time the experience was accessed, and those seeking to create a new group must attend a live information session before doing so.

The strengths of Grove VR appear to be its accessibility and the potential to create custom peer support groups on any mental health topic. Importantly, given the desk-based nature of this review and potential ethical issues, neither the appraisers nor panel members requested to join peer support groups on Grove VR. Despite this, it was still possible to access the virtual environment in which groups took place (campfire) and explore most features without attending meetings. However, as Stage 1 appraisers never ventured beyond the virtual lobby, the scores for Grove VR were low on each of the four domains. In addition, panel members were unable to comment on the quality and suitability of pre-existing Grove VR support groups. Finally, despite a strict privacy policy, specific community standards and related features, self-disclosure on a platform like Grove VR does seem to pose higher risks for having information exposed compared to conventional peer support groups. This is only in part compensated by the fact that participation in online groups can be anonymous. Nonetheless, the concept of Grove VR was considered somewhat unique among the experiences retrieved and could have therapeutic value as a virtual equivalent of (therapist-led) peer support groups, particularly in the context of COVID-19, but also thereafter.

## Discussion

The data presented here provides a unique overview of the evolving landscape in relation to freely available VR experiences for mental health therapy. In doing so, the authors address some of the long-standing issues with the accessibility and use of VR content within routine mental healthcare.

In total, 50 unique VR experiences met the final inclusion criteria representing 2.7% of the sample retrieved during initial screening. While exposure-based content was categorised most often by reviewers during quality appraisal, the most clearly defined experiences relevant to mental health were those that focused on relaxation or meditation. These experiences often made an explicit and direct reference to mental health conditions, such as anxiety or low mood whereas exposure-based content was, at times, less explicit and more subjective. While this subjectivity is a potential weakness within the appraisal process, in terms of exploratory work such as this review, it does highlight potential opportunities for mental health practitioners in terms of creative and innovative uses of VR content.

The development of a quality appraisal tool as part of this study provided the standardisation and robustness of early reviews and enabled an aggregated score to be attached to each VR experience. Reliability analysis indicated a high level of internal consistency, which strengthens the rigor of results that were brought to the panel discussions and analyses. Initial appraisals clearly demonstrate the ability of freely available VR content to induce a physical or emotional response with over 40% rating their experience as either high or moderate. In addition, over half (52%) the experiences that met initial inclusion criteria achieved a high or moderate assets score. This suggests that there are a number of features inherent within these experiences that may be useful within therapeutic contexts, such as opportunities for live performance monitoring, the ability to interact with the environment, to control/limit exposure or engagement, and the ability to be used in tandem with other therapies.

Using the graded and synthesised material gathered from the quality appraisal exercise, the panel was encouraged to draw upon their own areas of expertise in determining potential therapeutic value—they were not expected (nor would it be appropriate) to determine the effectiveness of a VR experience to treat a mental health condition or issue. Similar to previous research, the VR experiences retrieved were viewed as additional tools that mental health practitioners may choose to use to support their current practice (Cieślik et al., 2020). VR content was thus viewed in the same context as other traditional therapeutic tools, such as still imagery, activity diaries, thought records, flipchart work and paired words tasks.

Of the 50 experiences discussed, the panel felt that the majority were only tangentially related to mental health and that their use within a therapeutic setting would only be feasible in a small number of cases. This was mostly true of VR experiences that were primarily developed for other purposes but had some content that could be used for phobia-based treatment (e.g. horror games that contained spiders or enclosed, dark spaces for the treatment of arachnophobia, claustrophobia or nyctophobia). In addition, it was also considered that these games were designed to induce fear and not to reduce it, so the experience would have to be heavily moderated by the therapist. For the 11 experiences that were ultimately selected, discussion centred on how the VR content could fit within pre-existing treatment protocols. For example, VR experiences that were potentially useful for SAD (REC Room, BeFearless) were viewed as early tools to increase engagement and acceptability as well as to conduct initial behavioural experiments within a cognitive model of SAD treatment. Parallels can be drawn here with Freeman et al. (2018) who used a similar cognitive approach in treatment of those with fear of heights using VR. For those focusing on low mood or stress reduction through relaxation and meditation (Liminal, Calm Place etc.), the protocols and components within mindfulness-based cognitive behavioural therapy and humanistic counselling were discussed and linked to key concepts and techniques, such as acceptance, attention restoration and focused breathing. Finally, there were also discussions regarding exposure-based content and the steps a behavioural therapist might take to help users overcome their fears through a process of habituation (e.g. Bridge Trek and Fear of Heights).

### Limitations

There are a number of limitations to the current study. First, the end-user perspective has not been taken into account, as the panel did not contain any clients receiving mental health therapy. We also did not consider organisational factors and focused solely on individual-level application and feasibility. Second, VR applications that were labelled as promising by the panel have may have value for practice, but their real world application and feasibility use was not assessed. Third, panellists’ appraisal scores are subjective: personal fears, experience or therapeutic biases may have influenced their assessment as well as their own professional background and clinical training. Fourth, while searches took place within the two most popular VR databases, the review does not cover the whole field of freely available VR. Finally, there is no evidence presented here that suggests the VR applications retrieved provide effective treatment, and further research as to the therapeutic benefit of any of the experiences would be required.

In conclusion, this review produced a novel approach to reviewing VR experiences, using combination of systematic reviewing and interdisciplinary consensus building to offer mental health professionals an overview of freely available and accessible VR content for use within routine care. Results show an abundance of freely available applications, a small minority of which show potential relevance for mental health practitioners. Discussion concerning the potential use of these applications did result in concrete suggestions on practical implementation within routine care. The importance of such information cannot be underestimated, as up until now, most efforts in this domain have mainly focused on providing evidence of effect of specific VR experiences. In contrast, there seems to be too little ‘technical and treatment protocol information within academic publications … to give therapists the confidence to implement these new approaches within their current practice’ (Best et al., 2020, p.3). Nevertheless, when seeking to include VR technology within mental health therapy, practitioners would do well to (1) consider current treatment protocols for the specified mental health condition and (2) sufficiently familiarize themselves with the technology, its potential applications and features (e.g. control over exposure content, ability to monitor progress or outcomes). Moreover, practitioners should (3) assess individual client suitability (e.g. symptom level, knowledge and skill in relation to VR). For each individual session, they should also (4) discuss the rationale for relying on VR technology with the client and obtain consent to participate.

It is also important to emphasise that applications with demonstrated effectiveness are not always readily available or acquire elaborate set-ups and specialist facilities to make full use of them. The current paper has therefore attempted to overcome this gap, by providing a detailed and nuanced account on how freely accessible VR content may be used to support routine mental health care. Nevertheless, if those in routine practice want to make use of the full potential of virtual reality, it will have to move beyond free applications and also towards more high-end setups. What will remain quintessential, even with more professional set-ups, is further research on how VR content can be fully utilised within routine practice. In the meantime, though, low-threshold, freely accessible applications are out there that pave the way by raising awareness and acceptance with practitioners, to ultimately adopt VR within routine practice and form part of the therapist’s wider toolkit.

## Declarations

### Research Involving Human and Animal Participants

As the research design was a scoping review and involved no human participants or confidential data, formal ethical approval was not required.
